# Efficacy and mechanism of Qianjinweijing Decoction for asthma: Integrating systematic review with meta-analysis and network pharmacology

**DOI:** 10.1097/MD.0000000000041317

**Published:** 2025-01-31

**Authors:** Limin Zhang, Jin Su, Xiaozheng Wu, Yunzhi Chen, Wen Li

**Affiliations:** a Guizhou University of Traditional Chinese Medicine, Guiyang, Guizhou, China.

**Keywords:** asthma, efficacy, mechanism of action, meta-analysis, network pharmacology, Qianjinweijing Decoction

## Abstract

**Background:**

Asthma seriously affects people’s survival and quality of life, causing a huge economic burden on society. Modern clinical use of Qianjinweijing Decoction (QJWJ) for the treatment of asthma has achieved good results. However, there is still a lack of research on its efficacy and mechanism of action. Therefore, the purpose of this study is to evaluate the efficacy of QJWJ in the treatment of asthma by systematic review and meta-analysis, and to explore its potential mechanism by network pharmacology.

**Methods:**

The meta-analysis was performed to search for studies published before May 2023 in 7 databases, and Revman 5.4 and R language softwares were used for analysis. Network pharmacology was based on open databases and softwares such as Cytoscape, Perl, Autoduck Vina, and R language.

**Results:**

A total of 14 studies were included, involving 1200 patients. The results of the meta-analysis showed that QJWJ could significantly improve the clinical efficacy of asthma patients compared with routine pharmacotherapy (risk ratio = 1.22, 95% CI [1.16, 1.28], *P* < .00001), enhance lung function, such as FEV_1_/FVC (mean difference [MD] = 5.63, 95% CI [1.45, 9.81], *P* = .008), FEV_1_% (MD = 5.03, 95% CI [4.32, 5.74], *P* < .00001), PEF (standardized mean difference = 1.37, 95% CI [1.03, 1.71], *P* < .00001), and increase traditional Chinese medicine syndrome score (MD = −2.50, 95% CI [−4.81, −0.19], *P* = .03). The results of network pharmacology suggested that the 4 traditional Chinese medicines in QJWJ included 35 active ingredients and 34 potential targets for the treatment of asthma. The core ingredients involved were stigmasterol, β-sitosterol, hederagenin, and gibberellin 7. The core targets were PTGS2, BCL2, and CASP3. The interaction pathway between QJWJ and asthma was mainly enriched in p53, cyclic guanosine monophosphate–protein kinase G, IL-17, and advanced glycation end products-receptor for advanced glycation end products signaling pathways. Molecular docking showed that the core ingredients had good binding activity with the core targets.

**Conclusion:**

QJWJ is effective in the treatment of asthma, and the therapeutic mechanism may be related to its regulation of inflammation, immunity, and apoptosis.

## 1. Introduction

Asthma is a common clinical respiratory disease characterized by chronic airway inflammation, which is formed under the combined action of heredity and environment. The main symptoms are dyspnea, wheezing, chest tightness, and persistent cough.^[1]^ According to statistics, there are 350 million asthma patients worldwide, whose management and control are seriously inadequate, especially in low-income and middle-income countries.^[2]^ Air pollution and exposure to allergens are the main risk factors.^[3]^ Currently, inhaled corticosteroids, leukotriene modulators, biological agents, and allergen immunotherapy are mainly used.^[4]^ Although these treatments are effective, more than one-third of the patients’ symptoms cannot be effectively controlled.^[5]^ This makes it particularly urgent to find new and more effective treatments.

The characteristics of multi-component, multi-target, and multi-pathway of traditional Chinese medicine compounds are gradually recognized.^[6]^ Qianjinweijing Decoction (QJWJ), from the *Gujinluyanfang*, which is mostly used to treat lung carbuncle. The whole prescription is composed of phragmites stem (later generations of doctors often use Lugen instead of phragmites stem),^[7]^ coix seed, semen persicae, and semen benincasae. The main effect is clearing the lung and resolving phlegm, removing blood stasis and pus. In the prescription, phragmites stem clears heat and eliminates carbuncle, coix seed, and semen benincasae infiltrate dampness and pus, semen persicae relieves cough and dispels phlegm. In modern clinical practice, QJWJ is mostly used in the treatment of asthma and has achieved good results. Based on this, QJWJ may have a positive therapeutic effect on asthma. However, as a traditional Chinese medicine prescription, QJWJ has complex components, and its mechanism of action needs to be further studied. Therefore, this study uses meta-analysis to conduct a more comprehensive quantitative analysis of its efficacy, uses network pharmacology to predict its potential mechanism of action, and uses molecular docking to verify the results, in order to provide a precise and effective treatment for asthma.

## 2. Materials and methods

### 2.1. Meta-analysis

This part was performed in strict accordance with the PRISMA international standard,^[8]^ and registered on PROSPERO (https://www.crd.york.ac.uk/PROSPERO/), the registration number is CRD42023416916.

#### 2.1.1. Data sources and searches

Seven databases including CNKI, Wangfang, VIP, SinoMed, Pubmed, Embace, and Cochrane Library were searched by computer. Combined literature review with manual retrieval of subject words and free words, the Chinese search words were: “Qianjin Weijing Decoction” or “Weijing Decoction” and “asthma”; the key words were “asthma,” “Asthmas,” “Bronchial Asthma,” “Asthma, Bronchial.” The retrieval time was from the establishment of each database to May 2023. PubMed search strategy is shown in Table S1, Supplemental Digital Content, http://links.lww.com/MD/O284.

#### 2.1.2. Inclusion criteria

(1) Randomized controlled trials of QJWJ in the treatment of asthma and there is no restriction on the use of blinding. (2) Patients with asthma were included, with clear diagnostic criteria and efficacy evaluation criteria. There were no restrictions on the patient’s gender, age, race, and nationality. (3) Intervention measures: the experimental group: QJWJ was used on the basis of routine pharmacotherapy, or only QJWJ was used. QJWJ and its addition and subtraction and reed root instead of reed stem were included. Control group: routine pharmacotherapy. The dose, method of administration, and course of treatment of the 2 groups were not limited.

#### 2.1.3. Exclusion criteria

(1) Inclusion of non-asthmatic patients or interventions without QJWJ or non-randomized controlled trials would be excluded. (2) Studies whose full text were unavailable or data was missing. (3) For the same data from the same author, the most complete one of the data is retained.

#### 2.1.4. Results

Main outcome: clinical efficiency. Secondary outcomes: lung functions such as FEV_1_/FVC, FEV_1_%, and PEF; and TCM syndrome score.

#### 2.1.5. Studies screening and data extraction

This study was screening by 2 researchers (L.Z. and J.S.) independently. For the literature with differences, through discussion and resolution, those could not be resolved were handed over to the author Wen Li to determine whether to include. For studies without detailed results or lack of information, the authors were contacted by e-mail.

Data extraction mainly includes author, journal, year, subject characteristics, intervention measures, control design, outcome indicators, research duration, and research design.

#### 2.1.6. Assessment of bias risk

The Cochrane Collaboration’s 5.4 bias risk assessment tool was used for evaluation. The evaluation was carried out according to the scoring rules of “high risk,” “unclear,” and “low risk.”

#### 2.1.7. Statistical analysis

Revman5.4 software was used for forest plot production and subgroup analysis. R language software was used for sensitivity analysis, drawing funnel plot, and detecting publication bias. And Egger test was performed if necessary. The dichotomous variables were expressed as risk ratio (RR), the mean difference (MD) was used to represent continuous variables and standardized mean difference (SMD) was used to represent the data of the same outcome and different measurement units. The 95% confidence interval (95% CI) was calculated. The data analysis method was selected according to the heterogeneity of the included studies: when *P* > .10, *I*^2^ < 50%, the fixed effect model (FE) was selected; when *P* ≤ .10, *I*^2^ > 50%, the random effect (RE) was selected. Explore sources of heterogeneity when necessary. Descriptive analysis was performed when necessary. Publication bias test was performed on the data of ≥ 10 included studies.

### 2.2. Network pharmacology analysis

#### 2.2.1. Screening of active compounds and targets of QJWJ

The Traditional Chinese Medicine Systems Pharmacology Database and Analysis Platform (https://old.tcmsp-e.com/tcmsp.php) was used to search the active compounds of phragmites stem, coix seed, semen persicae, and semen benincasae in QJWJ. According to the results, oral bioavailability ≥ 30 % and drug-likeness ≥ .18 were used as the limiting conditions for screening. Perl (V5.30.0) was used to correspond the active compounds of traditional Chinese medicine to the corresponding targets 1 by 1. The gene symbol was obtained by using the Uniprot, and the repetition was deleted by Perl software and the target ID conversion of traditional Chinese medicine was performed.

#### 2.2.2. Identification of asthma targets

Using “asthma” as the search term, this study searched GeneCards (https://www.genecards.org/), PharmGkb (https://www.pharmgkb.org/), DrugBank (https://go.drugbank.com), OMIM (https://omim.org/), and TTD (http://db.idrblab.net/ttd/) to obtain asthma-related targets. R language software was used to delete duplicates and merge the genes from the above-mentioned databases to obtain disease targets. By mapping drugs and diseases 1 by 1, the intersection targets were obtained.

#### 2.2.3. Construction of “drug–ingredients–target” network for the treatment of asthma by QJWJ

Cytoscape software (V3.8.0) was used to construct the “drug–ingredients–target” network for the intersection targets of Section 2.2.2.

#### 2.2.4. Protein–protein interaction and core target screening

Substitute the intersection target of Section 2.2.2. into the String online platform (https://stringdb.org/) to construct the protein–protein interaction (PPI) network. CytoScape software was used to screen the core genes in the network. And the following 6 indicators of betweenness centrality, closeness centrality, degree centrality, Eigenvector centrality, local average centrality, and network centrality were calculated. Taking the condition that each indicator was greater than the median value at the same time as the screening condition, the final core target was obtained after 2 screenings.

#### 2.2.5. GO functional analysis and KEGG pathway enrichment analysis

Gene ontology (GO) functional enrichment analysis and Kyoto Encyclopedia of Genes and Genomes (KEGG) pathway enrichment analysis were performed on the intersection targets obtained by Section 2.2.2. using R language software and Bioconductor bioinformatics software package, and visualized.

#### 2.2.6. Molecular docking

Molecular docking of core components and key targets was performed. Download the 2D structure of active pharmaceutical ingredients on PubChem website (https://pubchem.ncbi.nlm.nih.gov/), and its 3D structure was obtained using Chemoffice (V14.0.0.117) software. Afterwards, the 3D structure of the key target was downloaded from the PDB database (http://www.rcsb.org/). The 3D structure of the key target was optimized by PyMol (2.4.0) software. Autodock Vina (V1.5.6) and PyMol software were used for molecular docking.

## 3. Results

### 3.1. Meta-analysis

#### 3.1.1. Data sources, searches results

**Figure 1.**
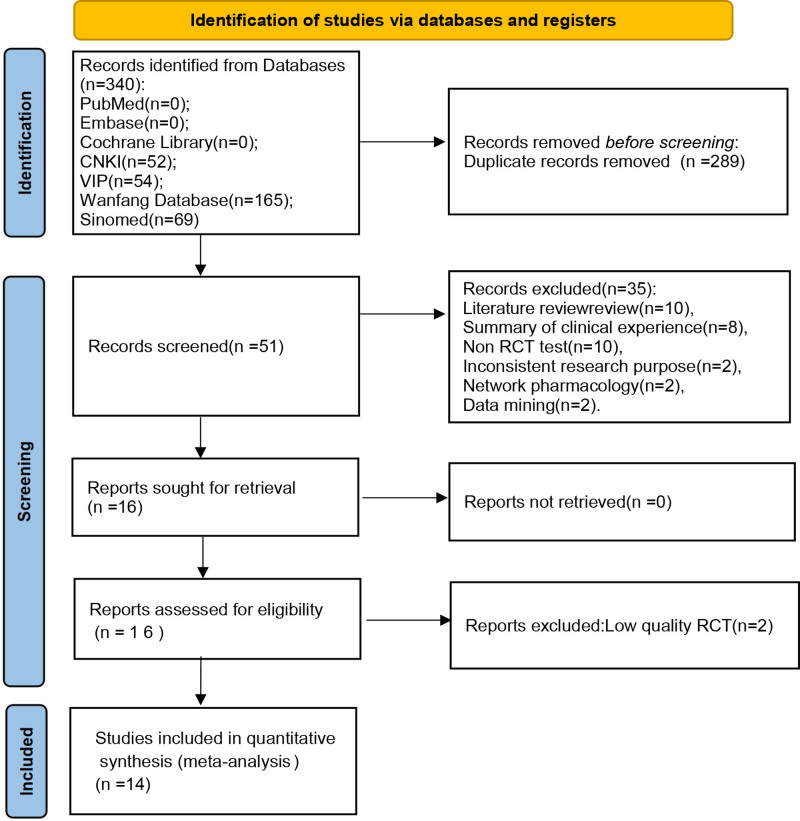
Flow chart.

A total of 340 related studies were retrieved. After eliminating duplication, reading the title, abstract and full text, and eliminating the studies that did not meet the inclusion criteria. Fourteen studies were finally included,^[9–22]^ all of which were Chinese studies. The screening process is indicated in Figure 1. The included studies involved 1200 patients, including 619 in the QJWJ group and 581 in the RP group. The basic characteristics are shown in Table 1.

**Table 1:** Basic characteristics of included studies.

Studies	Year	Gender (male/female)		Mean age		Number of patients		Intervention		Course of treatment (d)	Outcomes
		E	C	E	C	E	C	E	C		
Chen^[10]^	2020	16/15	17/14	51.42 ± 4.26	51.39 ± 4.18	31	31	RP + QJWJ combined Dingchuantang	RP	10	I, III, IV, V
Chen^[11]^	2017	17/13	16/14	52.3 ± 3.8	50.9 ± 3.9	30	30	RP + QJWJ combined Dingchuantang	RP	10	I, III
Cong^[12]^	2018	21/17	23/15	52.4 ± 11.6	52.9 ± 11.4	38	38	RP + QJWJ combined Dingchuantang	RP	10	I, III, V
Gao^[13]^	2011	38/22	39/21	60.2 ± 4.8	59.6 ± 5.6	60	60	RP + QJWJ	RP	30	I
Jin^[14]^	2016	20/14	19/15	38 ± 2.36	39 ± 2.51	34	34	QJWJ combined Dingchuantang	RP	10	I, II, IV
Ji^[15]^	2021	27/20	25/22	53.42 ± 8.71	53.63 ± 8.15	47	47	RP + QJWJ combined Dingchuantang	RP	10	III, IV
Ke^[16]^	2018	28/20	26/22	51.76 ± 8.05	52.12 ± 8.94	48	48	RP + QJWJ combined Dingchuantang	RP	10	I
Li^[17]^	2020	34/24	39/31	54.62 ± 5.94	55.98 ± 6.21	58	70	RP + QJWJ combined Dingchuantang	RP	10	I, II
Li^[18]^	2019	29/26	30/25	50.35 ± 5.45	50.54 ± 5.43	55	55	RP + QJWJ combined Dingchuantang	RP	10	I, II, IV
Luo^[19]^	2006	NA	NA	NA	NA	78	30	Weijingshengmaitang	RP	6	I, IV
Qiao^[20]^	2016	21/19	20/18	49.13 ± 3.87	51.87 ± 7.36	40	38	Jiaweiweijingtang	Chinese patent medicines	21	I
Song^[21]^	2014	16/18	17/17	51.12 ± 10.23	49.35 ± 9.75	34	34	RP + QJWJ combined Dingchuantang	RP	10	I, III, V
Wu^[22]^	2020	15/15	16/14	45.21 ± 1.01	45.15 ± 1.23	30	30	RP + QJWJ combined Dingchuantang	RP	10	I, III
Zheng^[23]^	2019	17/19	16/20	50.52 ± 6.46	50.76 ± 6.55	36	36	RP + QJWJ combined Dingchuantang	RP	10	I, III, IV

C = control group; E = experimental group; NA = not mentioned; QJWJ = Qianjinweijing Decoction; RP = routine pharmacotherapy; I = Clinical efficiency; II = FEV1/FVC; III = FEV1%; IV = PEF; V = TCM symptom score.

#### 3.1.2. Studies quality evaluation

**Figure 2.**
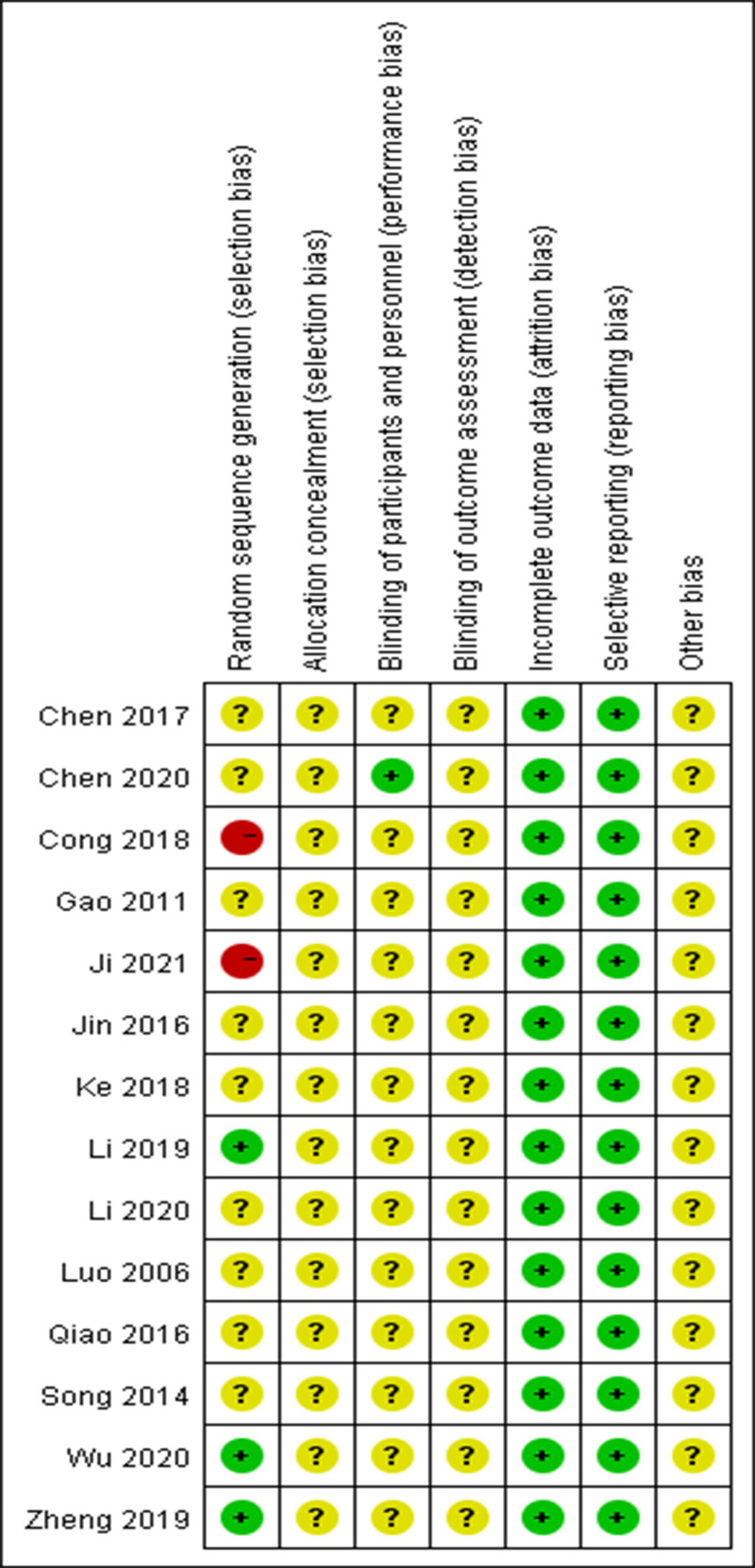
Risk-of-bias assessment.

Three studies^[17,21,22]^ described randomization methods and were rated as “low risk,” 2 studies^[11,14]^ grouped according to treatment methods and were rated as “high risk,” studies that only mentioned randomization were rated as “unclear.” The included studies did not mention the blind method of result evaluation and were rated as “unclear.” One study^[9]^ described blinding and was rated as “low risk,” while the remaining studies did not mention it and was rated as “unclear.” All studies without setting whether the result evaluation was blinded were rated as “unclear.” All the results were complete and the included studies had no reporting bias and were rated as “low risk.” No other bias was described and rated as “unclear.” The evaluation of the literature is presented in Figure 2.

#### 3.1.3. Clinical efficacy

**Figure 3.**
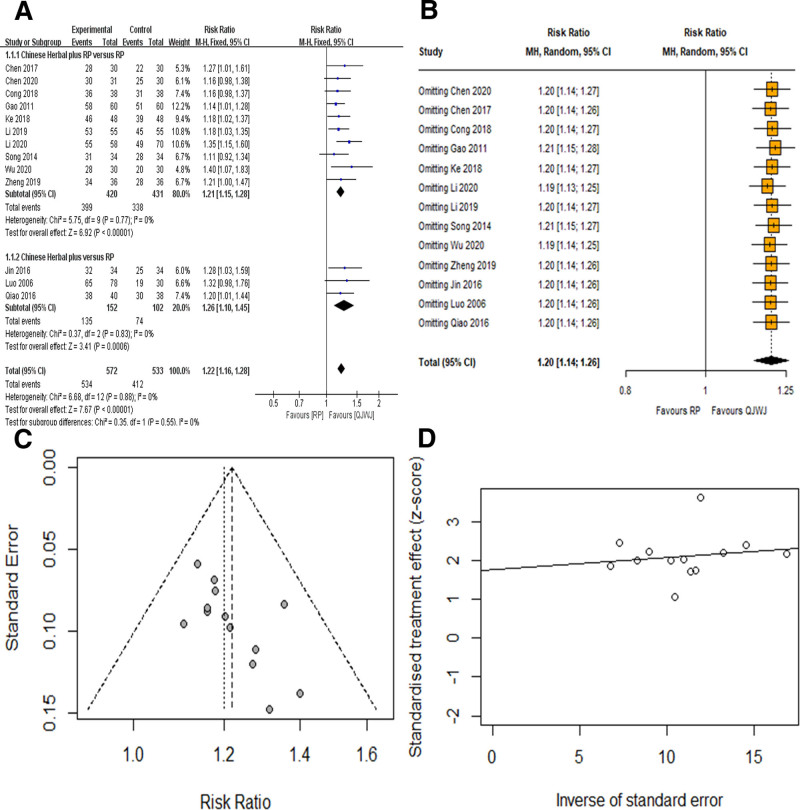
Clinical efficacy. (A) Clinical efficacy forest plot. (B) Sensitivity analysis plot. (C) Funnel plot. (D) Egger test.

Thirteen studies^[9–13,15–22]^ reported clinical efficacy. Heterogeneity test showed no heterogeneity (*I*^2^ = 0%, *P* = .88), so FE was selected. The results indicated that QJWJ could significantly improve the clinical efficacy of asthma patients [RR = 1.22, 95% CI (1.16, 1.28), *P* < .00001]. According to whether the control design was combined with routine pharmacotherapy, it was divided into 2 subgroups: Chinese Herbal plus RP versus RP and Chinese Herbal versus RP. The QJWJ group showed better clinical efficacy in the subgroup of Chinese Herbal plus RP versus RP [RR = 1.21, 95% CI (1.15, 1.28), *P* < .00001]. In the subgroup of Chinese Herbal versus RP, the QJWJ group also showed better clinical efficacy than the RP group [RR = 1.26, 95% CI (1.10, 1.45), *P* = .0006] (Fig. 3A). The results of sensitivity analysis showed that the results of deleting any of the studies were statistically significant (Fig. 3B). Observing the funnel plot, it was found that the distribution on both sides was asymmetric, suggesting the existence of publication bias (Fig. 3C). Egger test results showed that *P* = .0244, suggesting the existence of publication bias (Fig. 3D).

#### 3.1.4. Pulmonary function

##### 3.1.4.1. FEV_1_/FVC

**Figure 4.**
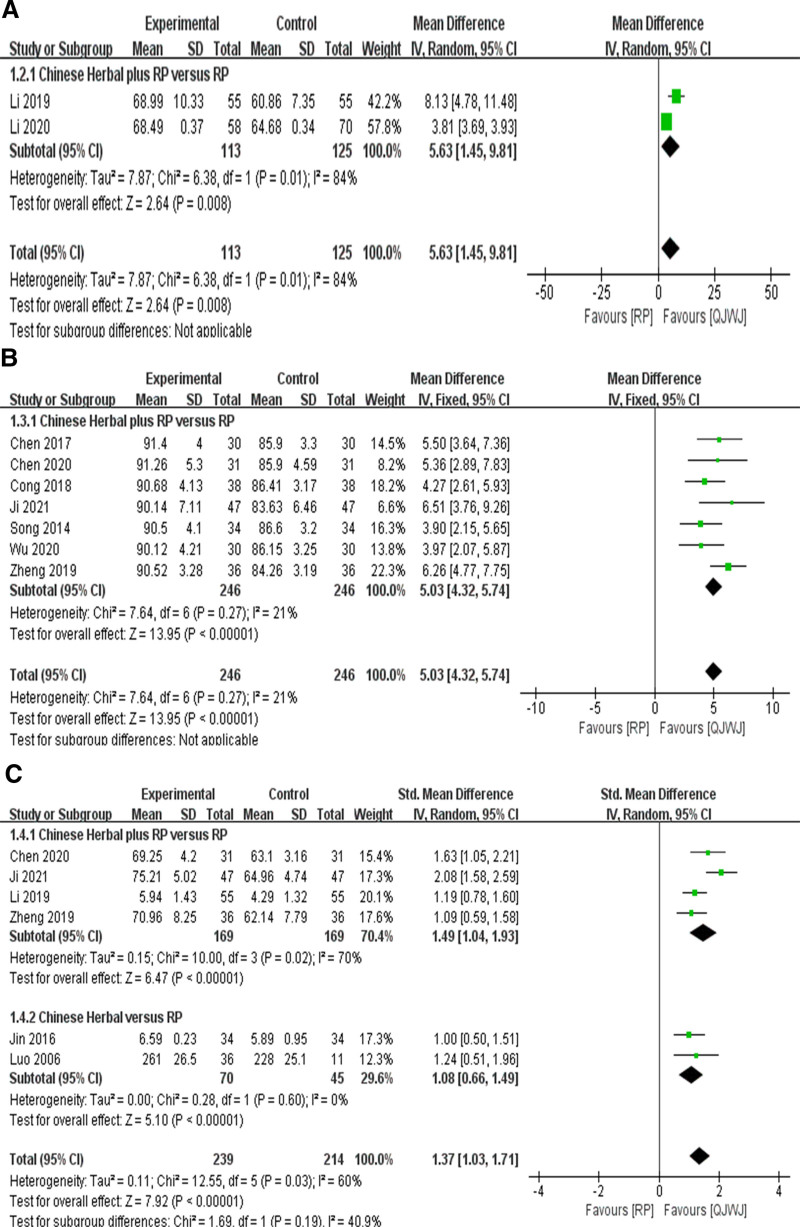
Lung function. (A) FEV1/FVC forest plot; (B) FEV1 % forest plot; (C) PEF forest plot.

Two studies^[16,17]^ reported FEV1/FVC with great heterogeneity (*I*^2^ = 84%, *P* = .01), so RE was selected. The control design of the 2 studies was Chinese Herbal plus RP versus RP. QJWJ group had a better improvement effect on FEV_1_/ FVC [MD = 5.63, 95% CI (1.45, 9.81), *P* = .008] (Fig. 4A).

##### 3.1.4.2. FEV_1_%

Seven studies^[9–11,14,20–22]^ reported FEV_1_%, and the heterogeneity was acceptable (*I*^2^ = 21%, *P* = .27), so FE was selected. The control design of the 7 studies was Chinese Herbal plus RP versus RP. The results indicated that the QJWJ group could significantly improve the FEV_1_% of the patients, which was better than the RP group (MD = 5.03, 95% CI [4.32, 5.74], *P* < .00001) (Fig. 4B).

##### 3.1.4.3. PEF

Six studies^[9,13,14,17,18,22]^ reported PEF, and the heterogeneity was large (*I*^2^ = 60%, *P* = .03), so RE was selected. The QJWJ group had a better improvement effect on PEF in patients with asthma (SMD = 1.37, 95% CI [1.03, 1.71], *P* < .00001). In order to analyze the source of heterogeneity, it was divided into 2 subgroups: Chinese Herbal plus RP versus RP and Chinese Herbal versus RP according to whether the control design was combined with routine pharmacotherapy. In the subgroup of Chinese Herbal plus RP versus RP, the improvement of PEF in the QJWJ group was more significant (SMD = 1.49, 95% CI [1.04, 1.93], *P* < .00001). In the subgroup Chinese Herbal versus RP, QJWJ group can significantly improve PEF in patients with asthma (SMD = 1.08, 95% CI [0.66, 1.49], *P* < .00001) (Fig. 4C). The results of sensitivity analysis indicated that after deleting the study of Ji,^[14]^ the heterogeneity was significantly reduced (*I*^2^ = 0%, *P* = .57), indicating that this study was the cause of the greater heterogeneity.

#### 3.1.5. TCM symptom score

**Figure 5.**
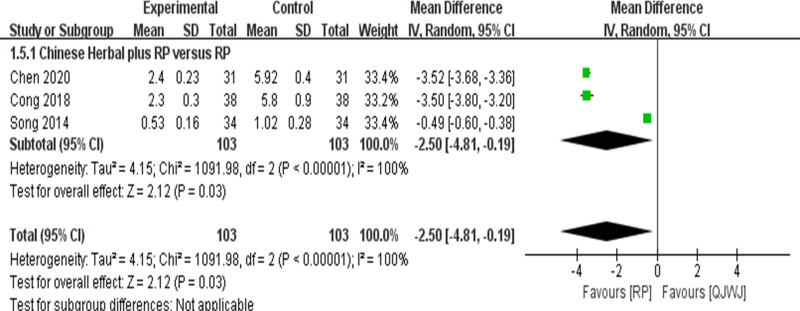
TCM symptom score forest plot.

Three studies^[9,11,20]^ reported TCM symptom scores, and the heterogeneity among the 3 studies was very large (*I*^2^ = 100%, *P* < .00001), so RE was selected. The control design of the 3 studies was Chinese Herbal plus RP versus RP. The results indicated that the improvement of TCM symptoms in the QJWJ group was better (MD = −2.50, 95% CI (−4.81, −0.19), *P* = .03] (Fig. 5). Sensitivity analysis showed that the heterogeneity was significantly reduced after deleting the study of Song^[20]^ (*I*^2^ = 0%, *P* = .91), indicating that this study was the reason for the large heterogeneity.

### 3.2. Network pharmacology analysis

#### 3.2.1. Screening results of active ingredients and targets of QJWJ

Through The Traditional Chinese Medicine Systems Pharmacology Database and Analysis Platform database, 35 active ingredients were obtained, including phragmites stem (1 ingredient), semen benincasae (2 ingredient), semen persicae (23 ingredient), and coix seed (9 ingredient). A total of 252 target genes of active ingredients were obtained, including 31 in phragmites stem, 34 in semen benincasae, 139 in semen persicae, and 48 in coix seed. The gene symbol was obtained by using the Uniprot, and 48 drug targets were obtained by using Perl software to delete the repetition and ID conversion. The main active ingredients of QJWJ are list in Table S2, Supplemental Digital Content, http://links.lww.com/MD/O284.

#### 3.2.2. Results of asthma target screening

**Figure 6.**
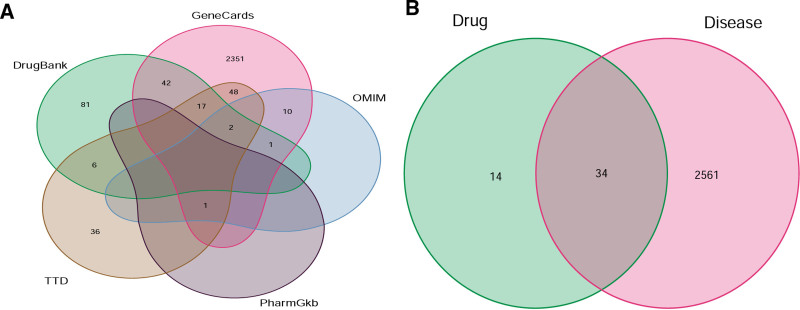
Venn diagram. (A) Venn diagram of asthma target. (B) Venn diagram of drug–disease intersection target.

With “asthma” as the key word, 5 public databases were searched respectively, and 3022 asthma-related gene targets were obtained: 2472 Gene Cards, 25 OMIM, 1 PharmGkb, 99 TTD, and 425 DrugBank. After merging and deleting the repeats, 2595 disease-related targets were obtained (Fig. 6A). By mapping 48 drug target genes with disease target genes 1 by 1, 34 target genes of QJWJ acting on asthma were obtained (Fig. 6B).

#### 3.2.3. QJWJ treatment of asthma regulatory network construction results

**Figure 7.**
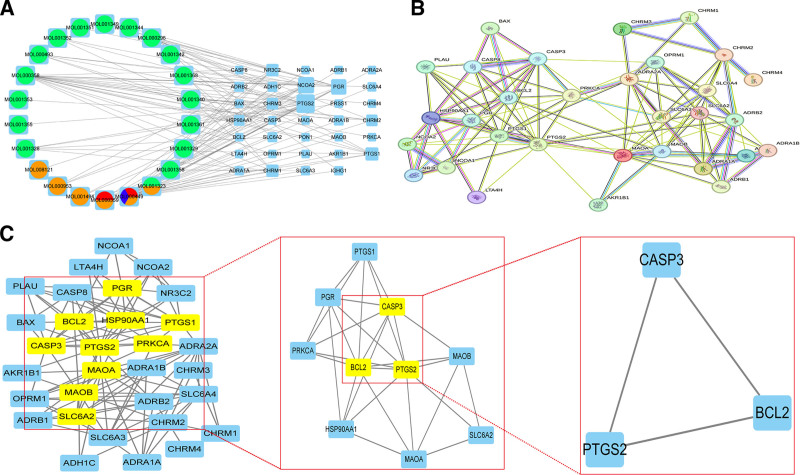
(A) “Drug–ingredients–target” network (green represents semen persicae, orange represents coix seed, red represents semen benincasae, and blue represents phragmites stem); (B) PPI network; (C) PPI network core screening. PPI = protein–protein interaction.

The “drug–ingredients–target” network was obtained by Cytoscape software (Fig. 7A). Degree values were sorted by software “network analyze” function, and 4 core ingredients were screened out: stigmasterol, β-sitosterol, hederagenin, and gibberellin 7.

#### 3.2.4. Results of PPI network construction and core target screening

Using String database to build PPI network. Medium confidence (.400) was selected, and 2 free segments were deleted to obtain the PPI network. The network consists of 33 nodes and 117 edges. Nodes represent proteins and edges represent interactions between proteins (Fig. 7B). The TSV file obtained when constructing the PPI network was imported into Cytoscape to construct a new network. Subsequently, the CytoNCA plug-in was used to extract betweenness centrality, closeness centrality, degree centrality, Eigenvector centrality, network centrality, and local average centrality greater than the median value to screen core genes. The 2 screening conditions are shown in the Table S3, Supplemental Digital Content, http://links.lww.com/MD/O284. Finally, 3 protein nodes were selected as candidate genes, including PTGS2, CASP3, and BCL2. These 3 key genes are the core targets of QJWJ in treating asthma (Fig. 7C).

#### 3.2.5. GO and KEGG enrichment analysis

**Figure 8.**
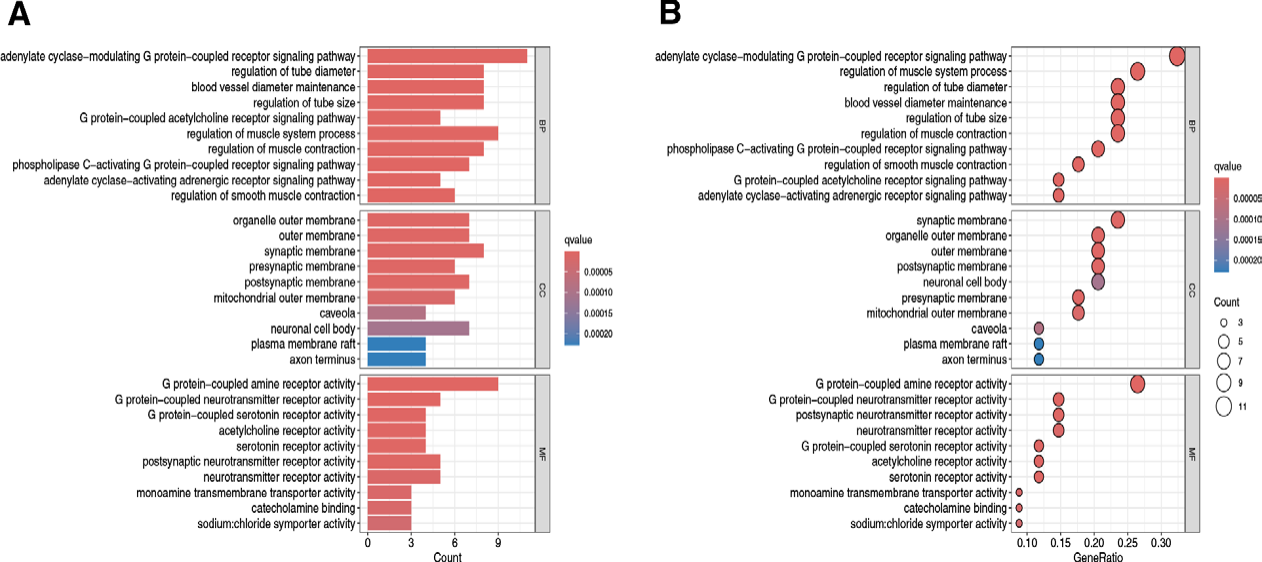
GO functional enrichment analysis. GO = gene ontology.

**Figure 9.**
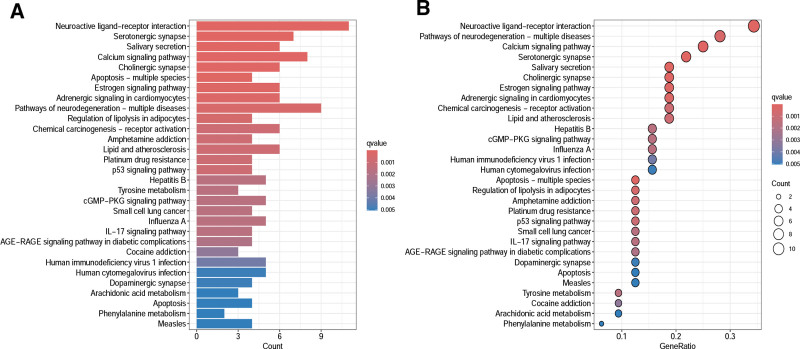
KEGG pathway enrichment analysis. KEGG = Kyoto Encyclopedia of Genes and Genomes.

A total of 840 GO functional enrichment analysis results were obtained, including 676 biological processes, 57 cellular components, and 109 molecular functions. The first 10 biological processes with obvious enrichment were shown in Figure 8. The KEGG pathway enrichment analysis showed that QJWJ played a role in asthma through p53 signaling pathway, cyclic guanosine monophosphate–protein kinase G (cGMP–PKG) signaling pathway, IL-17 signaling pathway, and advanced glycation end products–receptor for advanced glycation end products (AGE–RAGE) signaling pathway in diabetic complications. The top 30 pathways with obvious enrichment of KEGG pathway were shown in Figure 9.

#### 3.2.6. Molecular docking results

**Figure 10.**
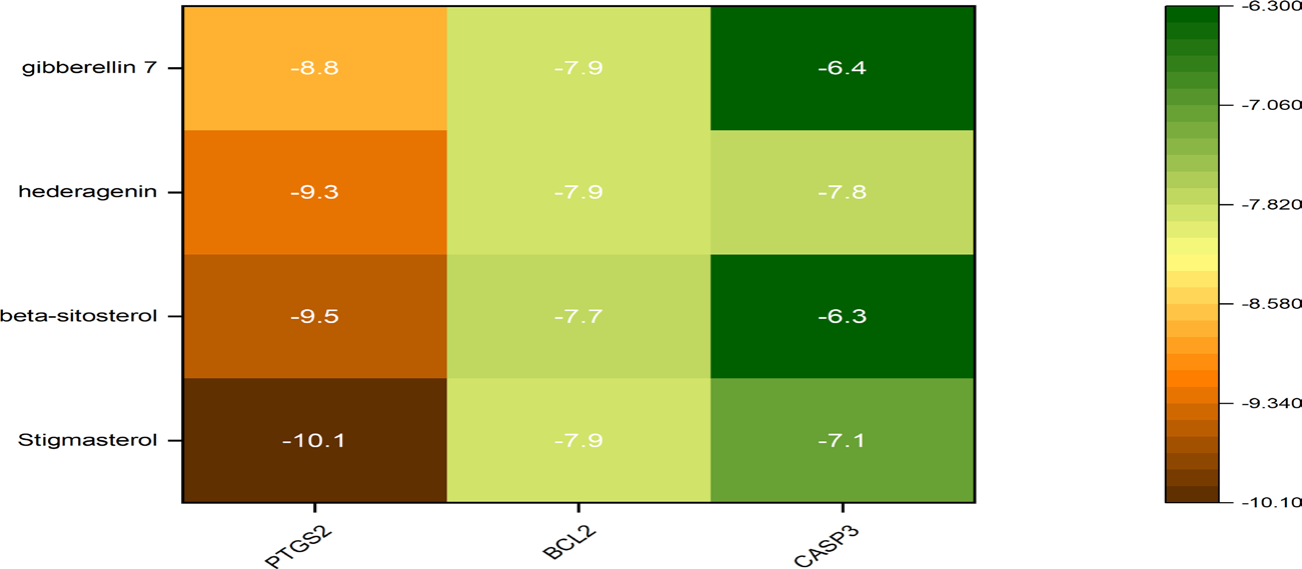
Binding energy heat map of core active components and core targets.

**Figure 11.**
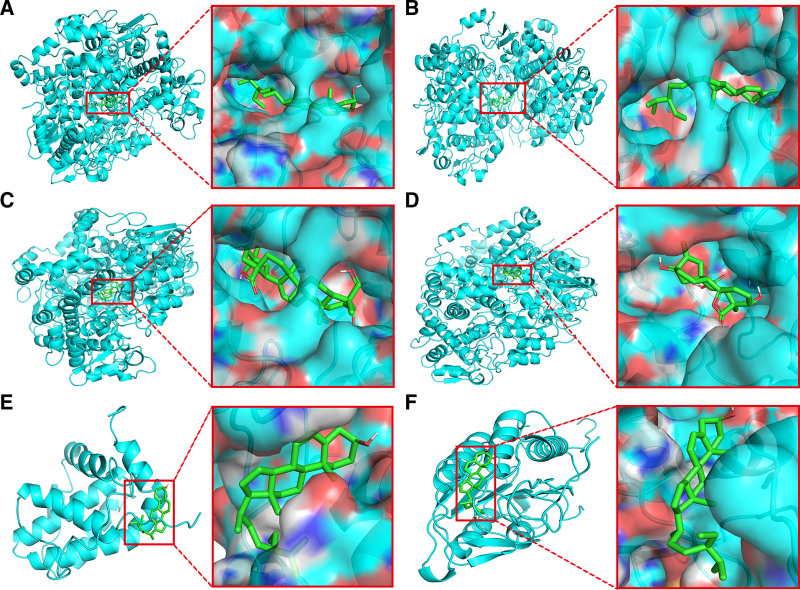
Visualization results of molecular docking. (A) PTGS2–stigmasterol; (B) PTGS2–β-sitosterol; (C) PTGS2–hederagenin; (D) PTGS2–gibberellin 7; (E) BCL2–β-sitosterol; (F) CASP3–β-Sitosterol.

In QJWJ, stigmasterol, β-sitosterol, hederagenin, and gibberellin 7 were selected as candidate docking pharmacodynamic ingredients, and key targets PTGS2, BCL2, and CASP3 were selected as candidate docking targets for molecular docking. The molecular docking thermal energy diagram was indicated in Figure 10, and the docking result unit is kal·mol^−1^. The molecular docking simulation diagram was constructed using PyMol software (Fig. 11). The results showed that stigmasterol, β-sitosterol, hederagenin, and gibberellin 7 had good binding activity with 3 key targets of asthma.

## 4. Discussion

The pathogenesis of asthma mainly involves inflammatory response, immune and allergic reactions, genetic phenotypes, etc. And inflammatory response is one of the key factors.^[1,23–25]^ However, due to its complex pathogenesis, the effectiveness of existing treatment options needs to be improved. Studies have found that QJWJ has a good therapeutic effect on asthma, but the research on its mechanism of action was not sufficient due to the complexity of its ingredients.

The meta-analysis part of this study analyzed its effectiveness. The study demonstrated that QJWJ alone or combined with routine pharmacotherapy provided better clinical efficacy in treating asthma compared to routine pharmacotherapy alone. In addition, the use of QJWJ alone or in combination with routine pharmacotherapy can significantly improve the level of lung function indexes such as FEV_1_/FVC, FEV_1_ % and PEF, and the related TCM syndromes of asthma have improved significantly, the effect is better than routine pharmacotherapy treatment.

Network pharmacology analysis was conducted to explore the mechanism of QJWJ in treating asthma based on meta-analysis. The results indicated that QJWJ contained 35 active ingredients and 34 potential targets for the treatment of asthma. Traditional Chinese medicine exhibits characteristics of multiple active ingredients and targets. By constructing the “drug–ingredients–target” network, stigmasterol, β-sitosterol, hederagenin, and gibberellin 7 are the core ingredients of the network. Stigmasterol and β-sitosterol are common sterol compounds with strong anti-inflammatory and anti-oxidative stress effects. Studies have shown that stigmasterol reduces the expression of IL-2 by inhibiting NK13-R, thereby reducing the inflammatory response and oxidative stress levels in asthmatic mice.^[26,27]^ Wang et al have proved that β-sitosterol can decrease the levels of TNF-α, IL-6, and TGF-β1, thereby improving the inflammation-related symptoms of asthmatic rat models.^[28]^ Hederagenin is a pentacyclic triterpenoid saponin with anti-inflammatory and antioxidant effects.^[29,30]^ Research studies have shown that hederagenin can help alleviate lung injury by inhibiting the activation of NF-κB signaling pathway, which in turn can inhibit the activation of NLRP3 inflammatory and M1 macrophages and reduce the inflammatory response.^[31]^

PPI network analysis showed that PTGS2, BCL2, and CASP3 were the key predictive targets of QJWJ in treating asthma. PTGS2 is an inflammation-inducing enzyme that is mainly involved in the inflammatory response.^[32]^ Studies have shown that inhibition of PTGS2 can reduce inflammation through the NF-κB signaling pathway, thereby potentially treating asthma.^[33]^ BCL2 is a key factor in the mitochondrial apoptosis pathway. By inhibiting the release of cytochrome C from mitochondria, it makes the downstream related cascade reaction unable to be activated and inhibits apoptosis, thereby reducing the inflammatory cells in the lungs of asthmatic mice, reducing the inflammatory response and alleviating asthma attacks.^[34,35]^ CASP3 is an important protease of the Caspase family, which can reflect the apoptosis of bronchial epithelial cells. The activation of Caspase-3 can inhibit apoptosis, maintain cell homeostasis, and slow down airway remodeling in asthma.^[36,37]^

The molecular docking results indicate reliable binding activity between the ligand and receptor. According to GO and KEGG enrichment analysis, QJWJ may regulate apoptosis and inflammatory response through p53 signaling pathway, cGMP–PKG signaling pathway, IL-17 signaling pathway, AGE–RAGE signaling pathway, and play a role in asthma inflammation and airway remodeling. Studies have shown that p53 can inhibit TORC1 activity, promote PTEN-mediated autophagy, and inhibit ERK1/2 signaling, playing an autophagy role in airway remodeling in asthma.^[38,39]^ cGMP–PKG activation can lead to BK_Ca_ channel opening, reduce PKG dependence, so that the airway relaxation, relieve asthma symptoms in guinea pigs.^[40]^ cGMP–PKG can also affect the phosphorylation level of Smad3, reduce the thickening of airway basement membrane, inhibit the process of airway epithelial–mesenchymal transition, and reduce the airway remodeling of asthma.^[41]^ IL-17 is a cytokine produced by Th17 cells, which is used to protect the surface tissues of the intestinal tract, gums, skin and other barriers.^[42]^ IL-17 is a driving factor in the immunopathology of asthma. Increased expression of IL-17 can be detected in lung tissue, sputum and serum of asthmatic patients.^[43]^ Advanced glycation end products–RAGE, a member of the cell surface receptor immunoglobulin superfamily, has the highest expression in the lungs and is a driver of inflammation. AGEs are its ligand, which binds to RAGE through NF-κB signaling and up-regulates pro-inflammatory mediators, leading to a variety of lung diseases, including asthma.^[44]^

This study has some limitations: (1) some of the included literature did not use blinding and allocation concealment; (2) some of the included studies were not rigorous in the use of random methods, and did not describe specific methods; (3) there are few studies on the use of QJWJ alone, which is not conducive to the comparison of its efficacy; (4) no adverse reactions were reported, and its safety could not be evaluated.

## 5. Conclusion

This study analyzed the efficacy of QJWJ in treating asthma and examined its potential mechanism. The results showed that combined with QJWJ or QJWJ alone had better clinical efficacy than conventional asthma drugs. The results of network pharmacology showed that stigmasterol, β-sitosterol, hederagenin, and gibberellin 7 were the core active ingredients of QJWJ, which may play a role through PTGS2, BCL2, and CASP3 targets, mainly involving p53 signaling pathway, cGMP–PKG signaling pathway, IL-17 signaling pathway, AGE–RAGE signaling pathway. The mechanism of QJWJ in treating asthma may be through the regulation of apoptosis, inflammation and immune response, and then play an active role in the inflammation, immune process, and airway remodeling of asthma.

## Acknowledgments

The authors thank the support of the National Natural Science Foundation of China [grant number 81760841], Rolling Support for Provincial Universities’ Scientific Research Platform Team Project of Guizhou Provincial Department of Education, Project No.: Guizhou Education Technology [2022]-023, and Guizhou University of Traditional Chinese Medicine for this study.

## Author contributions

**Conceptualization:** Limin Zhang.

**Data curation:** Limin Zhang, Jin Su.

**Formal analysis:** Limin Zhang.

**Funding acquisition:** Yunzhi Chen.

**Investigation:** Limin Zhang.

**Methodology:** Limin Zhang, Wen Li.

**Project administration:** Wen Li.

**Resources:** Limin Zhang, Jin Su, Wen Li.

**Software:** Limin Zhang, Jin Su.

**Supervision:** Xiaozheng Wu, Yunzhi Chen, Wen Li.

**Validation:** Limin Zhang, Jin Su, Xiaozheng Wu, Yunzhi Chen, Wen Li.

**Writing – original draft:** Limin Zhang.

**Writing – review & editing:** Jin Su, Xiaozheng Wu, Yunzhi Chen, Wen Li.

## Supplementary Material


