# Supplementary material for: Efficacy and mechanism of Qianjinweijing Decoction for asthma: Integrating systematic review with meta-analysis and network pharmacology
Source: Medicine (Baltimore). 2025 Jan 31;104(5):e41317. doi: 10.1097/MD.0000000000041317 (PMC11789889; doi:10.1097/MD.0000000000041317)
Supplement: Supplementary file 1 [file medi-104-e41317-s001.docx]

Table S1.

| Number | Search Terms |
| --- | --- |
| #1 | Mesh descriptor: (asthma) explode all fields |
| #2 | (“asthma”[Title/Abstract] OR“Asthmas”[Title/Abstract] OR“Bronchial Asthma”[Title/Abstract] OR “Asthma, Bronchial”[Title/Abstract]) |
| #3 | Or1-2 |
| #4 | Mesh descriptor: (weijing decoction) explode all trees |
| #5 | "weijing decoction"[Title/Abstract] OR "Phragmites Stem Decoction"[Title/Abstract] |
| #6 | Or 4-5 |
| #7 | 3 and 6 |

PubMed search strategy.

Table S2.

200 main active ingredients of QJWJ.

| Drug | MolId | MolName | Symbol |
| --- | --- | --- | --- |
| Dongguazi | MOL000359 | sitosterol | PGR |
| Dongguazi | MOL000359 | sitosterol | NCOA2 |
| Dongguazi | MOL000359 | sitosterol | NR3C2 |
| Dongguazi | MOL000449 | Stigmasterol | PGR |
| Dongguazi | MOL000449 | Stigmasterol | NR3C2 |
| Dongguazi | MOL000449 | Stigmasterol | NCOA2 |
| Dongguazi | MOL000449 | Stigmasterol | ADH1C |
| Dongguazi | MOL000449 | Stigmasterol | IGHG1 |
| Dongguazi | MOL000449 | Stigmasterol | RXRA |
| Dongguazi | MOL000449 | Stigmasterol | NCOA1 |
| Dongguazi | MOL000449 | Stigmasterol | PTGS1 |
| Dongguazi | MOL000449 | Stigmasterol | PTGS2 |
| Dongguazi | MOL000449 | Stigmasterol | ADRA2A |
| Dongguazi | MOL000449 | Stigmasterol | SLC6A2 |
| Dongguazi | MOL000449 | Stigmasterol | SLC6A3 |
| Dongguazi | MOL000449 | Stigmasterol | ADRB2 |
| Dongguazi | MOL000449 | Stigmasterol | AKR1B1 |
| Dongguazi | MOL000449 | Stigmasterol | PLAU |
| Dongguazi | MOL000449 | Stigmasterol | LTA4H |
| Dongguazi | MOL000449 | Stigmasterol | MAOB |
| Dongguazi | MOL000449 | Stigmasterol | MAOA |
| Dongguazi | MOL000449 | Stigmasterol | CTRB1 |
| Dongguazi | MOL000449 | Stigmasterol | CHRM3 |
| Dongguazi | MOL000449 | Stigmasterol | CHRM1 |
| Dongguazi | MOL000449 | Stigmasterol | ADRB1 |
| Dongguazi | MOL000449 | Stigmasterol | SCN5A |
| Dongguazi | MOL000449 | Stigmasterol | ADRA1A |
| Dongguazi | MOL000449 | Stigmasterol | CHRM2 |
| Dongguazi | MOL000449 | Stigmasterol | ADRA1B |
| Dongguazi | MOL000449 | Stigmasterol | GABRA1 |
| lugen | MOL000449 | Stigmasterol | PGR |
| lugen | MOL000449 | Stigmasterol | NR3C2 |
| lugen | MOL000449 | Stigmasterol | NCOA2 |
| lugen | MOL000449 | Stigmasterol | ADH1C |
| lugen | MOL000449 | Stigmasterol | IGHG1 |
| lugen | MOL000449 | Stigmasterol | RXRA |
| lugen | MOL000449 | Stigmasterol | NCOA1 |
| lugen | MOL000449 | Stigmasterol | PTGS1 |
| lugen | MOL000449 | Stigmasterol | PTGS2 |
| lugen | MOL000449 | Stigmasterol | ADRA2A |
| lugen | MOL000449 | Stigmasterol | SLC6A2 |
| lugen | MOL000449 | Stigmasterol | SLC6A3 |
| lugen | MOL000449 | Stigmasterol | ADRB2 |
| lugen | MOL000449 | Stigmasterol | AKR1B1 |
| lugen | MOL000449 | Stigmasterol | PLAU |
| lugen | MOL000449 | Stigmasterol | LTA4H |
| lugen | MOL000449 | Stigmasterol | MAOB |
| lugen | MOL000449 | Stigmasterol | MAOA |
| lugen | MOL000449 | Stigmasterol | CTRB1 |
| lugen | MOL000449 | Stigmasterol | CHRM3 |
| lugen | MOL000449 | Stigmasterol | CHRM1 |
| lugen | MOL000449 | Stigmasterol | ADRB1 |
| lugen | MOL000449 | Stigmasterol | SCN5A |
| lugen | MOL000449 | Stigmasterol | ADRA1A |
| lugen | MOL000449 | Stigmasterol | CHRM2 |
| lugen | MOL000449 | Stigmasterol | ADRA1B |
| lugen | MOL000449 | Stigmasterol | GABRA1 |
| Taoren | MOL001323 | Sitosterol alpha1 | PGR |
| Taoren | MOL001323 | Sitosterol alpha1 | PTGS2 |
| Taoren | MOL001323 | Sitosterol alpha1 | GABRA1 |
| Taoren | MOL001323 | Sitosterol alpha1 | ADH1C |
| Taoren | MOL001323 | Sitosterol alpha1 | NR3C2 |
| Taoren | MOL001328 | 2,3-didehydro GA70 | PTGS1 |
| Taoren | MOL001328 | 2,3-didehydro GA70 | CHRM1 |
| Taoren | MOL001328 | 2,3-didehydro GA70 | PTGS2 |
| Taoren | MOL001328 | 2,3-didehydro GA70 | SLC6A2 |
| Taoren | MOL001328 | 2,3-didehydro GA70 | GABRA1 |
| Taoren | MOL001328 | 2,3-didehydro GA70 | PRSS1 |
| Taoren | MOL001328 | 2,3-didehydro GA70 | GRIA2 |
| Taoren | MOL001329 | 2,3-didehydro GA77 | PTGS2 |
| Taoren | MOL001329 | 2,3-didehydro GA77 | CA2 |
| Taoren | MOL001329 | 2,3-didehydro GA77 | GABRA1 |
| Taoren | MOL001329 | 2,3-didehydro GA77 | NCOA2 |
| Taoren | MOL001340 | GA120 | CHRM3 |
| Taoren | MOL001340 | GA120 | CHRM1 |
| Taoren | MOL001340 | GA120 | PTGS2 |
| Taoren | MOL001340 | GA120 | CHRM2 |
| Taoren | MOL001340 | GA120 | GABRA1 |
| Taoren | MOL001342 | GA121-isolactone | PGR |
| Taoren | MOL001344 | GA122-isolactone | PGR |
| Taoren | MOL001349 | 4a-formyl-7alpha-hydroxy-1-methyl-8-methylidene-4aalpha,4bbeta-gibbane-1alpha,10beta-dicarboxylic acid | NR3C2 |
| Taoren | MOL001349 | 4a-formyl-7alpha-hydroxy-1-methyl-8-methylidene-4aalpha,4bbeta-gibbane-1alpha,10beta-dicarboxylic acid | PGR |
| Taoren | MOL001351 | Gibberellin A44 | NR3C2 |
| Taoren | MOL001351 | Gibberellin A44 | GABRA1 |
| Taoren | MOL001352 | GA54 | PTGS2 |
| Taoren | MOL001352 | GA54 | HSP90AA1 |
| Taoren | MOL001352 | GA54 | NCOA2 |
| Taoren | MOL001352 | GA54 | CAMTA2 |
| Taoren | MOL001353 | GA60 | CHRM2 |
| Taoren | MOL001353 | GA60 | GABRA1 |
| Taoren | MOL001353 | GA60 | GRIA2 |
| Taoren | MOL001355 | GA63 | PTGS2 |
| Taoren | MOL001355 | GA63 | GABRA1 |
| Taoren | MOL001355 | GA63 | GRIA2 |
| Taoren | MOL001358 | gibberellin 7 | CHRM3 |
| Taoren | MOL001358 | gibberellin 7 | CHRM1 |
| Taoren | MOL001358 | gibberellin 7 | PTGS2 |
| Taoren | MOL001358 | gibberellin 7 | SLC6A3 |
| Taoren | MOL001358 | gibberellin 7 | ADRB2 |
| Taoren | MOL001358 | gibberellin 7 | SLC6A4 |
| Taoren | MOL001360 | GA77 | GABRA1 |
| Taoren | MOL001360 | GA77 | GRIA2 |
| Taoren | MOL001361 | GA87 | PTGS2 |
| Taoren | MOL001361 | GA87 | CA2 |
| Taoren | MOL001368 | 3-O-p-coumaroylquinic acid | PTGS1 |
| Taoren | MOL001368 | 3-O-p-coumaroylquinic acid | PTGS2 |
| Taoren | MOL001368 | 3-O-p-coumaroylquinic acid | HSP90AA1 |
| Taoren | MOL001368 | 3-O-p-coumaroylquinic acid | NCOA2 |
| Taoren | MOL001368 | 3-O-p-coumaroylquinic acid | CAMTA2 |
| Taoren | MOL000296 | hederagenin | PGR |
| Taoren | MOL000296 | hederagenin | NCOA2 |
| Taoren | MOL000296 | hederagenin | CHRM3 |
| Taoren | MOL000296 | hederagenin | CHRM1 |
| Taoren | MOL000296 | hederagenin | CHRM2 |
| Taoren | MOL000296 | hederagenin | ADRA1B |
| Taoren | MOL000296 | hederagenin | GABRA1 |
| Taoren | MOL000296 | hederagenin | GRIA2 |
| Taoren | MOL000296 | hederagenin | IGHG1 |
| Taoren | MOL000296 | hederagenin | ADH1B |
| Taoren | MOL000296 | hederagenin | ADH1C |
| Taoren | MOL000296 | hederagenin | LYZL6 |
| Taoren | MOL000296 | hederagenin | PTGS1 |
| Taoren | MOL000296 | hederagenin | SCN5A |
| Taoren | MOL000296 | hederagenin | PTGS2 |
| Taoren | MOL000296 | hederagenin | RXRA |
| Taoren | MOL000296 | hederagenin | SLC6A2 |
| Taoren | MOL000358 | beta-sitosterol | PGR |
| Taoren | MOL000358 | beta-sitosterol | NCOA2 |
| Taoren | MOL000358 | beta-sitosterol | PTGS1 |
| Taoren | MOL000358 | beta-sitosterol | PTGS2 |
| Taoren | MOL000358 | beta-sitosterol | HSP90AA1 |
| Taoren | MOL000358 | beta-sitosterol | KCNH2 |
| Taoren | MOL000358 | beta-sitosterol | DRD1 |
| Taoren | MOL000358 | beta-sitosterol | CHRM3 |
| Taoren | MOL000358 | beta-sitosterol | CHRM1 |
| Taoren | MOL000358 | beta-sitosterol | SCN5A |
| Taoren | MOL000358 | beta-sitosterol | CHRM4 |
| Taoren | MOL000358 | beta-sitosterol | ADRA1A |
| Taoren | MOL000358 | beta-sitosterol | CHRM2 |
| Taoren | MOL000358 | beta-sitosterol | ADRA1B |
| Taoren | MOL000358 | beta-sitosterol | ADRB2 |
| Taoren | MOL000358 | beta-sitosterol | CHRNA2 |
| Taoren | MOL000358 | beta-sitosterol | SLC6A4 |
| Taoren | MOL000358 | beta-sitosterol | OPRM1 |
| Taoren | MOL000358 | beta-sitosterol | GABRA1 |
| Taoren | MOL000358 | beta-sitosterol | BCL2 |
| Taoren | MOL000358 | beta-sitosterol | BAX |
| Taoren | MOL000358 | beta-sitosterol | CASP9 |
| Taoren | MOL000358 | beta-sitosterol | CASP3 |
| Taoren | MOL000358 | beta-sitosterol | CASP8 |
| Taoren | MOL000358 | beta-sitosterol | PRKCA |
| Taoren | MOL000358 | beta-sitosterol | PON1 |
| Taoren | MOL000358 | beta-sitosterol | MAP2 |
| Taoren | MOL000493 | campesterol | PGR |
| Taoren | MOL000493 | campesterol | PTGS1 |
| Taoren | MOL000493 | campesterol | PTGS2 |
| Taoren | MOL000493 | campesterol | HSP90AA1 |
| Taoren | MOL000493 | campesterol | NCOA2 |
| Yiyiren | MOL001323 | Sitosterol alpha1 | PGR |
| Yiyiren | MOL001323 | Sitosterol alpha1 | PTGS2 |
| Yiyiren | MOL001323 | Sitosterol alpha1 | GABRA1 |
| Yiyiren | MOL001323 | Sitosterol alpha1 | ADH1C |
| Yiyiren | MOL001323 | Sitosterol alpha1 | NR3C2 |
| Yiyiren | MOL001494 | Mandenol | PTGS1 |
| Yiyiren | MOL001494 | Mandenol | PTGS2 |
| Yiyiren | MOL001494 | Mandenol | NCOA2 |
| Yiyiren | MOL000359 | sitosterol | PGR |
| Yiyiren | MOL000359 | sitosterol | NCOA2 |
| Yiyiren | MOL000359 | sitosterol | NR3C2 |
| Yiyiren | MOL000449 | Stigmasterol | PGR |
| Yiyiren | MOL000449 | Stigmasterol | NR3C2 |
| Yiyiren | MOL000449 | Stigmasterol | NCOA2 |
| Yiyiren | MOL000449 | Stigmasterol | ADH1C |
| Yiyiren | MOL000449 | Stigmasterol | IGHG1 |
| Yiyiren | MOL000449 | Stigmasterol | RXRA |
| Yiyiren | MOL000449 | Stigmasterol | NCOA1 |
| Yiyiren | MOL000449 | Stigmasterol | PTGS1 |
| Yiyiren | MOL000449 | Stigmasterol | PTGS2 |
| Yiyiren | MOL000449 | Stigmasterol | ADRA2A |
| Yiyiren | MOL000449 | Stigmasterol | SLC6A2 |
| Yiyiren | MOL000449 | Stigmasterol | SLC6A3 |
| Yiyiren | MOL000449 | Stigmasterol | ADRB2 |
| Yiyiren | MOL000449 | Stigmasterol | AKR1B1 |
| Yiyiren | MOL000449 | Stigmasterol | PLAU |
| Yiyiren | MOL000449 | Stigmasterol | LTA4H |
| Yiyiren | MOL000449 | Stigmasterol | MAOB |
| Yiyiren | MOL000449 | Stigmasterol | MAOA |
| Yiyiren | MOL000449 | Stigmasterol | CTRB1 |
| Yiyiren | MOL000449 | Stigmasterol | CHRM3 |
| Yiyiren | MOL000449 | Stigmasterol | CHRM1 |
| Yiyiren | MOL000449 | Stigmasterol | ADRB1 |
| Yiyiren | MOL000449 | Stigmasterol | SCN5A |
| Yiyiren | MOL000449 | Stigmasterol | ADRA1A |
| Yiyiren | MOL000449 | Stigmasterol | CHRM2 |
| Yiyiren | MOL000449 | Stigmasterol | ADRA1B |
| Yiyiren | MOL000449 | Stigmasterol | GABRA1 |
| Yiyiren | MOL008121 | 2-Monoolein | NCOA2 |
| Yiyiren | MOL000953 | CLR | PGR |
| Yiyiren | MOL000953 | CLR | NR3C2 |
| Yiyiren | MOL000953 | CLR | NCOA2 |

Table 3.

| Parameter | Filter1 | Filter2 |
| --- | --- | --- |
| Betweenness centrality | 13.13636641 | 1.666666667 |
| Closeness centrality | 0.491803279 | 0.692307692 |
| Degree centrality | 8 | 5 |
| Eigenvector centrality | 0.16583389 | 0.295652986 |
| local average centrality | 3.555555556 | 4 |
| Network centrality | 4.666666667 | 5 |

Screening condition.
